# Benzene Metabolism Is Dominated by a High-Affinity Pathway at Ambient Exposures with Implications for Cancer Risks

**DOI:** 10.3390/ijms26178550

**Published:** 2025-09-03

**Authors:** Reuben Thomas, Sungkyoon Kim, Qing Lan, Roel Vermeulen, Luoping Zhang, Nathaniel Rothman, Martyn T. Smith, Stephen M. Rappaport

**Affiliations:** 1 J. David Gladstone Institutes, San Francisco, CA 94158, USA; reuben.thomas@gladstone.ucsf.edu; 2 Department of Environmental Health Sciences, Graduate School of Public Health, Seoul National University, Seoul 08826, Republic of Korea; ddram2@snu.ac.kr; 3 Occupational and Environmental Epidemiology Branch, Division of Cancer Epidemiology and Genetics, National Cancer Institute, National Institutes of Health, Department of Health and Human Service, Rockville, MD 20850, USA; qingl@mail.nih.gov (Q.L.); rothmann@mail.nih.gov (N.R.); 4 Division of Environmental Epidemiology, Institute for Risk Assessment Sciences, Utrecht University, 3508 TD Utrecht, The Netherlands; r.c.h.vermeulen@uu.nl; 5 School of Public Health, University of California, Berkeley, CA 94720, USA; luoping@berkeley.edu (L.Z.); martynts@berkeley.edu (M.T.S.)

**Keywords:** toxicokinetics, Michaelis-Menten models, high-affinity pathway, muconic acid, urinary benzene, weight of evidence, CYP2A13

## Abstract

Benzene is a ubiquitous environmental pollutant that induces blood cancers via its complex metabolism. Since cancer risks to the general public involve toxic benzene metabolites derived from the inhalation of benzene at ppb air concentrations, questions remain regarding low-dose metabolism. Using previously published data from 389 Chinese workers, we fit Michaelis–Menten-like models to predict urinary concentrations of *E*,*E*-muconic acid (the most discriminating urinary metabolite) as functions of urinary benzene levels between 0.0001 μM and 54 μM, equivalent to benzene air concentrations between 0.1 ppb and more than 100 ppm. When we compared models having either one or two metabolic pathways, weights of evidence favoring two pathways were essentially 100 percent for nonsmoking males and females and 58 percent for smoking males. At ppb exposure levels, metabolic rates for the high-affinity pathway were 43-fold greater than those for the low-affinity pathway in nonsmoking males, 6.5-fold greater in nonsmoking females, and 4.9-fold greater in smoking males. Thus, the high-affinity pathway is most efficient in nonsmoking males and is inhibited by smoking. The characteristics of the two-pathway model implicate lung metabolism of benzene via CYP2A13 and/or CYP2F1 at ppb air levels and liver metabolism by CYP2E1 above one ppm. Since ambient benzene concentrations are typically less than 10 ppb, blood-cancer risks predicted from workers exposed to above 1 ppm likely underestimate risks to the general public by many fold, and these risks may be modulated by smoking. Also, since the lung is the site of initial metabolism upon inhalation, the respiratory bioactivation of benzene could contribute to lung-cancer incidence, including that for lung adenomas in never smokers.

## 1. Introduction

**Figure 1:**
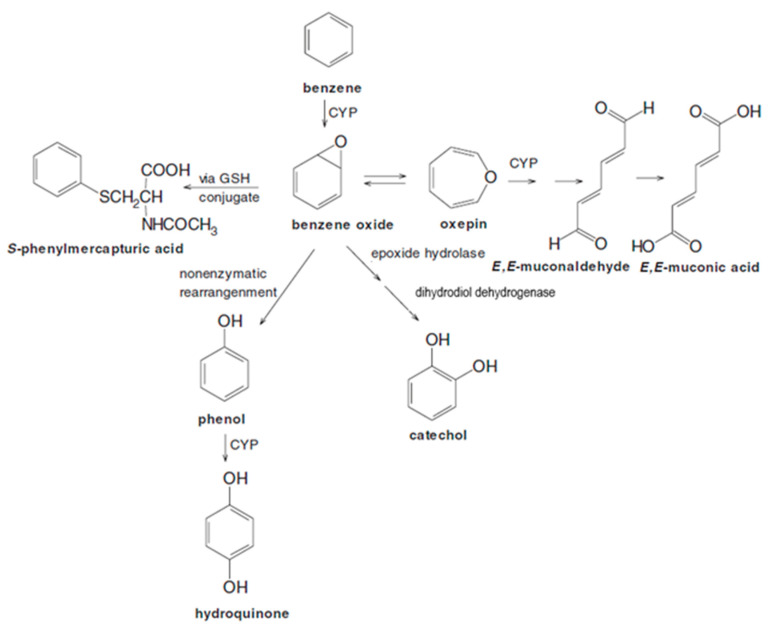
Simplified scheme of benzene metabolism showing prominent urinary metabolites. Abbreviations: CYP, cytochrome P450; GSH, glutathione. (This figure was modified from a more complex version published by the same authors [7], there is no need for copyright permission).

Benzene is an important industrial chemical and is a component of crude oil, gasoline, and combustion products, including cigarette smoke. This simple molecule is toxic to the blood and bone marrow (hematotoxicity) and induces various hematological malignancies [1], particularly myeloid neoplasms [2,3]. The carcinogenicity of benzene has been linked to its complex metabolism [4,5]. The initial metabolic step involves cytochrome P450 (CYP)-dependent oxidation (notably via CYP2E1 in the liver) of benzene to benzene oxide, which gives rise to a cascade of major metabolites (Figure 1) including phenolic products—phenol, hydroquinone, and catechol—and a ring-opened product—*E*,*E*-muconic acid (MA)—which collectively represent virtually all benzene metabolism [6].

Although much is known about benzene toxicity, key questions remain regarding human cancer risks posed by ambient exposures, such as those emanating from refineries, petrochemical complexes, natural gas fields, gasoline stations, engine exhausts, and combustion products including environmental tobacco smoke. Multiple studies have reported associations between ambient benzene exposures and the risk of various blood cancers, including childhood leukemia [8,9,10,11]; however, the mechanism(s) underlying these findings has been elusive. Because metabolism is necessary for benzene to exert hematotoxicity, and is governed by saturable kinetics, we have been investigating the dose-specific metabolism (DSM) of benzene, i.e., the concentrations of benzene metabolites per unit of exposure, for two decades. This line of inquiry was motivated by intriguing results from measurements of albumin adducts of reactive benzene metabolites that pointed to increased DSM at air concentrations below 1 ppm, which is well below the saturable range for CYP2E1 (>10 ppm) [12].

To investigate saturable metabolism, we measured the four major urinary benzene metabolites (phenol, MA, hydroquinone, and catechol) and a minor metabolite (*S*-phenylmercapturic acid) (see Figure 1) as well as levels of benzene in air and urine from 389 workers in factories that did and did not utilize benzene in Tianjin, China [7]. In this context, ‘control’ workers from factories without benzene were nonetheless exposed to this toxicant from ambient sources including active and passive smoking and reflect exposures to the general public. Because the air samplers used to measure airborne benzene had a detection limit of about 0.2 ppm, we had predicted lower air concentrations from measurements of urinary benzene (UB) and a calibration model, based on paired measurements of UB and air benzene (AB) above 0.2 ppm. Using general linear models we observed a 9-fold reduction in DSM (μM total benzene metabolites/ppm benzene) in the 389 subjects over the exposure range of 0.03 to 88.9 ppm and also found significant metabolic effects of sex, age, smoking status [13], and polymorphic forms of metabolizing genes [14]. Importantly, the DSM for major benzene metabolites was always greatest at benzene exposures of less than 1 ppm.

This finding prompted us to question whether a hitherto unrecognized high-affinity pathway was responsible for sub-ppm metabolism of benzene. To investigate this hypothesis, we employed an information-theoretic approach [15] to fit Michaelis–Menten-like (MM-like) models that included either one or two metabolic pathways to levels of AB and urinary metabolites from 263 nonsmoking females with exposures ranging from <0.001 to 299 ppm [16]. The results provided strong statistical evidence favoring two metabolic pathways for each of the major benzene metabolites and their sum (total urinary metabolites) in nonsmoking females. Under the two-pathway model, it was predicted that a nonsmoking Chinese female exposed to ppb levels of benzene would produce 0.184 µM total urinary metabolites per ppb of benzene, which is comparable to the prediction of 0.194 µM/ppb derived from independent data [16]. In fact, the putative high-affinity pathway accounted for about 70% of total urinary metabolites at air concentrations below 0.1 ppm in nonsmoking females. The low-affinity pathway accounted for most metabolite production at exposures above 1 ppm in nonsmoking females and primarily represented CYP2E1 metabolism, consistent with decreased productions of MA, phenol, and hydroquinone by homozygous individuals with variant CYP2E1 (rs2031920: C→T) compared with homozygous wild-types [14]. Attempts at including male subjects and smokers in the MM-like models of Chinese subjects were unsuccessful, in part, because most of the males and very few of the females smoked.

Price et al. used a request under the U.S. Freedom of Information Act (FOIA) to obtain data from our Tianjin study [13] and published a reanalysis of the data using the same spline regression models [17]. Although the authors reproduced our DSM findings, they questioned our use of UB to predict AB exposures below 0.2 ppm. This and other areas of disagreement—related largely to the adjustment for background metabolite levels—led to an exchange of published letters [18,19,20]. Subsequent reanalyses of the same data further questioned our use of UB to predict low AB exposures as well as our methods for estimating DSM, and suggested that routes of exposure other than inhalation, notably dermal contact, may have influenced the results [21,22]. Because these reanalyses of the Tianjin data limited their regressions to subjects with AB concentrations above the limit of detection (0.1–0.2 ppm), they excluded workers with exposures at ppb levels that were critical for low-dose-model fitting. Also, the analyses of McNally et al. [21] excluded subjects from one factory that used benzene because of co-exposures to toluene, which is also a substrate for CYP2E1. However, our previous regression modeling of the Tianjin data found no significant effect of co-exposure to toluene on urinary levels of any measured benzene metabolite [14]. Finally, none of these reanalyses of the Tianjin data explored the preliminary MM-like models that had bolstered our DSM findings.

### 1.1. Michaelis-Menten-like Models

Given questions related to prediction of sub-ppm benzene exposures from UB levels and possible routes of exposure other than inhalation, we report here a reanalysis of the original data from [7] to produce MM-like models that use UB levels as the exposure metric. Because UB was measured in all subjects and represents the internal dose of benzene regardless of the exposure route, i.e., inhalation, ingestion, or dermal contact, this approach reduces ambiguity in the interpretation of results. We used urinary MA as the outcome variable in our models because this benzene metabolite had low background concentrations in urine and was the most discriminating benzene metabolite at low exposure levels [7].

Motivated by [16,23], these MM-like models replaced the enzymatic velocity (*V*) with the concentration of urinary MA ([*MA*], μM), and replaced the substrate concentration with the UB concentration ([*UB*], μM). For subject *i*, each data pair ([*MA*]*_i_*, [*UB*]*_i_*) represents the respective urinary concentrations of MA and UB. MM-like kinetics based on one-pathway (Equation (1)) and two-pathway (Equation (2)) models for the metabolism of benzene to MA were fitted separately to data derived from female nonsmokers, male nonsmokers, and male smokers (analyses were confined to nonsmoking females because only eight of the females were smokers). Since the levels of [*MA*] and [*UB*] were both highly skewed and heteroscedastic, natural logarithms were used for regression analyses, employing the ‘transform-both-sides’ approach to stabilize the variance [24]. Under MM-like kinetics for the two models,

(1) $$ln{\left[MA\right]}_{i}=\ln\left({\left[MA\right]}_{0}^{\left(1\right)}+\frac{V_{max,1}^{\left(1\right)}{\left[UB\right]}_{i}}{\left(K_{m,1}^{\left(1\right)}+{\left[UB\right]}_{i}\right)}\right)+\varepsilon_{i}^{\left(1\right)},\;\mathrm{or}$$

(2) $$\ln{\left[MA\right]}_{i}=\ln\left({\left[MA\right]}_{0}^{\left(2\right)}+\frac{V_{max,1}^{\left(2\right)}{\left[UB\right]}_{i}}{\left(K_{m,1}^{\left(2\right)}+{\left[UB\right]}_{i}\right)}+\frac{V_{max,2}^{\left(2\right)}{\left[UB\right]}_{i}}{\left(K_{m,2}^{\left(2\right)}+{\left[UB\right]}_{i}\right)}\right)+\varepsilon_{i}^{\left(2\right)},\;\mathrm{for}$$

(3) $$\varepsilon_{i}^{\left(1\right)}\sim N\left(0,\sigma_{\left(1\right)}^{2}\right),\;\mathrm{and}$$

(4) $$\varepsilon_{i}^{\left(2\right)}\sim N\left(0,\sigma_{\left(2\right)}^{2}\right),$$

where$\;{\left[MA\right]}_{0}^{\left(1\right)}$ and ${\left[MA\right]}_{0}^{\left(2\right)}\;$denote the background levels of [*MA*] under the two models from sources other than benzene including sorbic acid [25]. Under Model 1, $V_{max,1}^{\left(1\right)}$ represents the asymptotically maximum level of [*MA*] and $K_{m,1}^{\left(1\right)}$ denotes the value of [*UB*] at which [*MA*]=$\;V_{max,1}^{\left(1\right)}$/2 for the one-pathway model of benzene metabolism. Under Model 2, $V_{max,1}^{\left(2\right)}$ and $K_{m,1}^{\left(2\right)}$ denote the corresponding kinetic parameters for the low-affinity pathway while $V_{max,2}^{\left(2\right)}$ and $K_{m,2}^{\left(2\right)}$ denote those for the high-affinity pathway. The respective error distributions were assumed to follow normal probability distributions (Equations (3) and (4)).

### 1.2. Linear and Generalized Additive Models (GAMs) for Benzene Metabolism

To provide a broader context for benzene metabolism, the MM-like Models 1 and 2 were augmented with linear models and Generalized Additive Models (GAMs) fit to the ln[*MA*]_i_ values as functions of the ln[*UB*]_i_ values. While the MM-like models provide a mechanistic interpretation of human benzene metabolism, the nonparametric GAMs allow the data to guide the profiles of the resulting best fits of [*MA*] to varying values of [*UB*], and the linear models enforce a simple parametric relationship that is often considered. Although neither the GAMs nor the linear models offer insight into a biological basis for the observed responses, comparisons of their fits with those from the MM-like models—based on the corresponding AIC values—allows one to assess the adequacy of Michaelis–Menten kinetics for interpreting the available data.

### 1.3. Weights of Evidence for Models

In judging the weights of evidence favoring the one- and two-pathway MM-like models (Models 1 and 2)—as well as the GAMs and linear models—as depictions of the true metabolism for benzene, we followed lines based on information theory [15] as described previously [16]. Using the difference in AIC values across any pair of the four candidate models [ΔAIC = (higher AIC) − (lower AIC)] permits estimation of the associated Akaike weight of evidence (*w*) for that comparison.

Regarding the two candidate MM-like models, the Akaike weight has a value of 1/(1 + exp(−ΔAIC/2)) ≤ *w* ≤ 1, indicating the weight of evidence supporting the model with a lower AIC as a better depiction of benzene metabolism. Similarly, Akaike weights comparing the two-parameter MM-like models to GAMs and linear models provide insight into the overall best fit.

Results from our models provide convincing evidence that a high-affinity, low-capacity metabolic pathway is responsible for benzene metabolism at ambient exposure levels and implicate the lung as the likely site of this metabolism.

## 2. Results

**Figure 2:**
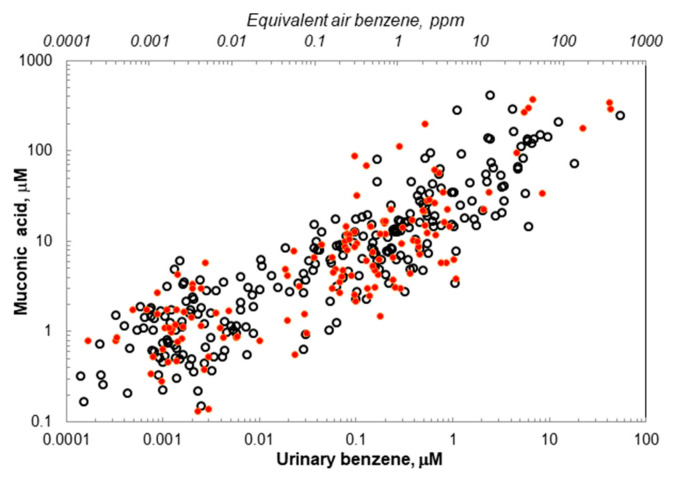
Scatter plot of 389 urinary benzene, *E*,*E*-muconic acid data pairs with open (black) and closed (red) markers representing females and males, respectively.

The data pairs ([*MA*]_i_, [*UB*]_i_) for the 389 subjects are given in Supplementary Materials along with subject identifiers: age, sex, exposure status, and smoking status. The scatter plot shown as Figure 2 displays a monotonic increase in [*MA*]_i_ with increasing [*UB*]_i_ from 0.0001 μM to 54 μM. For comparison with roughly equivalent AB concentrations, Figure 2 includes a secondary *X*-axis of $\left[AB\right]=exp\left[\frac{\ln(\left[UB\right]x1000)-5.42}{0.886}\right]$ from [7], where [*AB*] is given in ppm and [*UB*] in μM. Both male and female subjects were represented throughout the dynamic range of 0.0001 μM < [*UB*] < 54 μM, equivalent to [*AB*] values between 0.1 ppb and >100 ppm. (Because these equivalent values of [*AB*] were derived from a simple log-linear model using measured ([*AB*]_i_, [*UB*]_i_) data pairs for [*AB*]_i_ > 0.2 ppm, they tend to overestimate AB values in the saturable range above 100 ppm). The variation in [*MA*]_i_ at a given value of [*UB*]_i_ is approximately ten-fold.

When MM-like Models 1 and 2 were fit separately to the three sets of [*MA*]_i_ and [*UB*]_i_ data pairs, they converged with the fit statistics shown in Table 1. The value of the Model-2 AIC was much smaller than that of Model 1 for nonsmoking females (583.84 vs. 597.57) and males (148.59 vs. 156.34) but less so for smoking males (215.32 vs. 215.98). This resulted in Akaike weights of evidence favoring the two-pathway model with *w* = 1.00 and 0.98 in nonsmokers—indicating superior fits—and with *w* = 0.58 for male smokers—indicating a marginally better fit. When compared with the linear model, the two-pathway model provided superior fits with Akaike weights of *w* = 1.00 for female nonsmokers and male smokers and *w* = 0.89 for male nonsmokers. When compared with GAMs, the two-pathway model provided comparable fits with Akaike weights of 0.67 in female nonsmokers and 0.44 in male nonsmokers and a marginally worse fit in smoking males with an Akaike weight of 0.21. This indicates that the two-pathway model provides fits as good as or better than those of GAMs despite their comparative simplicity and strong biological basis. This conclusion is bolstered by figures in the Supplementary Materials comparing the two-pathway model predictions with those from GAMs for nonsmoking females (Figure S1) and nonsmoking and smoking males (Figures S2 and S3, respectively). In all three cases, the predictions from GAMs are largely contained within the 95% confidence intervals for the two-pathway models, even in low-dose regions with sparse data (Figures S2 and S3).

**Figure 3:**
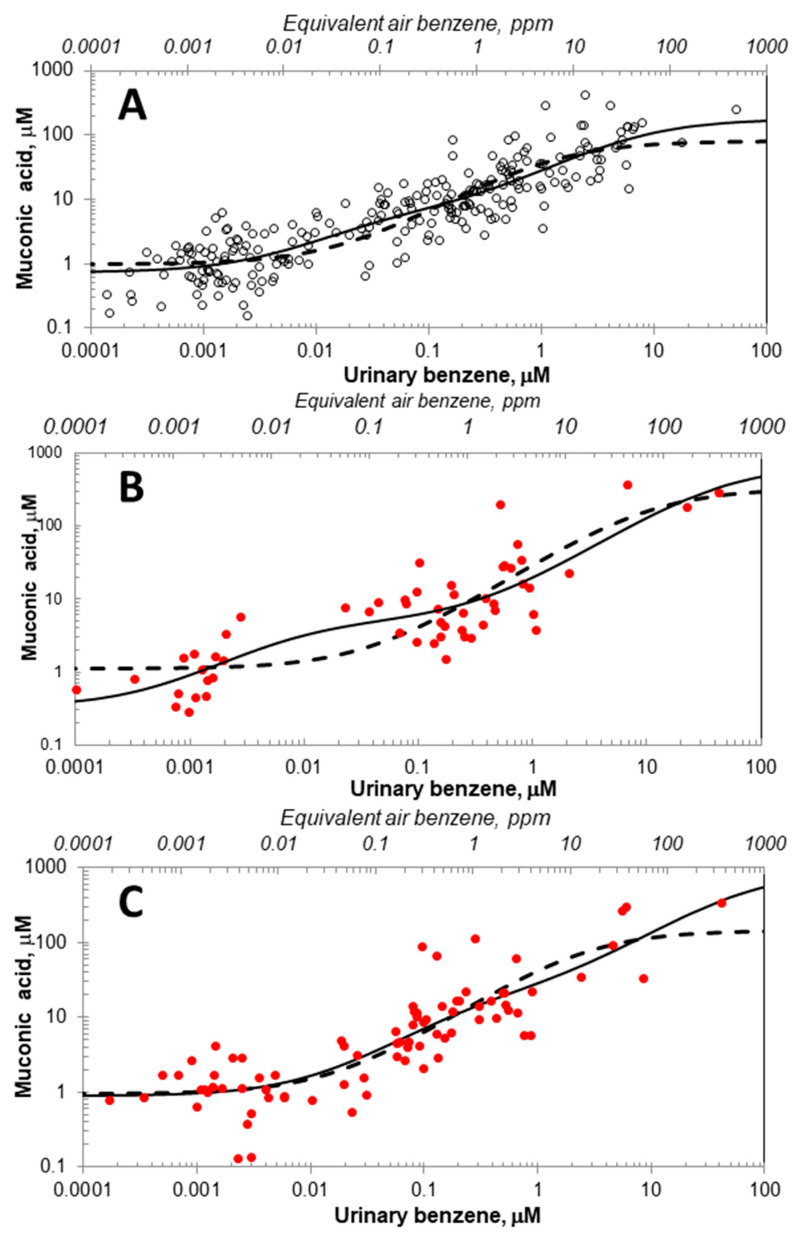
Scatter plots of urinary benzene and *E*,*E*-muconic acid data pairs for subsets of 243 female nonsmokers (**A**), 55 male nonsmokers (**B**), and 83 male smokers (**C**), and the corresponding fitted Michaelis–Menten-like Models 1 (dashed lines) and 2 (solid lines). (Females are represented by open black markers and males with closed red markers consistent with Figure 2).

Figure 3A–C show data for subjects stratified by sex and smoking status and the corresponding fitted one-pathway and two-pathway models based on the estimated parameters in Table 2, with dashed lines representing Model 1 and solid lines representing Model 2. Among nonsmokers, Figure 3A,B shows the pronounced increase in benzene metabolism under Model 2 relative to Model 1 in the low-dose range between 0.002 < [*UB*] < 0.1 μM, corresponding to 0.003 < [*AB*] < 0.4 ppm. Interestingly, this increased low-dose metabolism under Model 2 is greatly diminished in smoking males (Figure 3C).

## 3. Discussion

### 3.1. Updated Michaelis-Menten-like Models

After fitting MM-like Models 1 and 2 to the three Tianjin datasets, the weight of evidence supporting two metabolic pathways, i.e., Model 2, was essentially 100 percent in nonsmokers but only marginally greater than that of Model 1 in smoking males (Table 1). Our ability to characterize these dual metabolic pathways reflects the remarkable dynamic range of [*UB*], which covered more than six orders of magnitude (0.0001 to 54 μM). The superiority of Model 2 in the nonsmoking subjects is apparent in the scatter plots and corresponding fitted models in Figure 3A,B, which show that the one-pathway model (Model 1) tends to underestimate [*MA*] at [*UB*] values below 0.1 μM (equivalent to a predicted value of 0.4 ppm [*AB*]) and, to a lesser extent, in smoking males (Figure 3C). Indeed, evidence of saturation of the high-affinity pathway-two under Model 2 begins at about 0.02 μM [*UB*], while saturation of the low-affinity pathway-one does not begin until [*UB*] is about 500 times greater (10 μM).

Table 2 includes the 95% confidence intervals for the parameters of the respective models. In general, the width of these confidence intervals or the precision of the underlying estimates tracks with the number of subjects used to fit the models. So, the confidence interval for the model fit using the data of 243 female nonsmokers is the narrowest while that for the model fit to the 55 male nonsmokers is the widest. Despite the relatively wide confidence intervals for some of the individual model parameters, the 95% confidence intervals of the mean fitted [*MA*] responses (in logarithmic scale) allow one to observe significant dose-dependent responses for [*UB*] across the three models (Figures S1–S3), that is, the 95% confidence intervals of the mean-fitted [*MA*] levels at lower doses do not overlap those in the higher-dose range.

The largest sex- and smoking-related effects are observed for the high-affinity pathway, where *Rate 2* was almost 4-fold greater in nonsmoking males than nonsmoking females (642 vs. 165) and 10-fold greater for nonsmoking males than smoking males (642 vs. 66.2). In contrast, the low-affinity-pathway *Rate 1* was 70–90 percent greater in nonsmoking females (25.4) than either nonsmoking males (15.0) or smoking males (13.4). Although the limited number of nonsmoking males in the critical 1–10 ppb range prevents definitive conclusions about sex differences at ambient exposure levels, these differences in *Rate 1* and *Rate 2* across groups partially explain the roughly 10-fold range of variation in values of [*MA*]_i_ that was observed at a given value of [*UB*]_i_ (Figure 2). Unexplained sources of variation include random errors in measurements of UB and MA and the timing of urine sampling relative to the exposure regime. (Because UB is a highly transient biomarker, urine samples collected after the work shift can misrepresent the contributions of AB levels received earlier in the workday).

We focus now on the rate ratios, i.e., values of $\frac{Rate\;2}{Rate\;1}$ under Model 2 that represent the fold increases in the metabolism of benzene to MA via high-affinity pathway-two relative to low-affinity pathway-one in the low-dose linear range (where [*UB*]_i_ << $K_{m,2}^{\left(2\right)}$). As shown in Table 2, these rate ratios decrease from 42.7 for nonsmoking males to 6.50 for nonsmoking females and 4.94 for smoking males. This indicates that the high-affinity-pathway two is more active in nonsmoking males and is inhibited by smoking. In our previous MM-like model of MA versus airborne benzene exposures in 263 nonsmoking females [23], *Rate 1* = 7.38 and *Rate 2* = 53.9 μM MA per ppm AB, from which $\frac{Rate\;2}{Rate\;1}$ = 7.30, which is quite similar to the value of $\frac{Rate\;2}{Rate\;1}$ = 6.45 from our current model of [*MA*] versus [*UB*] in nonsmoking females. Furthermore, the estimated rate ratios for the two largest samples of subjects in the Tianjin study (6.50 in nonsmoking females, *N* = 243, and 4.94 in smoking males, *N* = 83) are comparable to the 5-fold decrease in DSM for all subjects at AB concentrations between 0.03 (DSM = 21.1 μM MA per ppm) and 89 ppm (DSM = 4.43 μM MA per ppm) that had been predicted in the Tianjin study [13].

Table 2 also shows estimates of the $Rate\;2\;proportion=\frac{Rate\;2}{Rate\;1+Rate\;2}\;$that represents the fraction of benzene metabolism to MA attributable to high-affinity-pathway two in the low-dose-linear range. This proportion varied between 0.832 for smoking males and 0.977 for nonsmoking males, indicating that between 83 and 98 percent of low-dose benzene metabolism resulted from high-affinity-pathway two. To the extent that MA is a good surrogate for benzene metabolism generally, these results suggest that virtually all benzene derived from ambient exposures is metabolized via high-affinity-pathway two. 

For context, the median benzene air concentration predicted from [*UB*] values for our nonsmoking control subjects was 4.34 ppb (*N* = 100) compared with measured ambient air concentrations from adult populations in the U.K. (3.82 ppb daytime mean value, *N* = 50) [26], Germany (3.44 ppb median value, *N* = 113) [27], and the U.S. (2.29–7.01 ppb range of median values for five cities, *N* = 421) [28]. Comparing these ambient air concentrations with the equivalent air-benzene concentrations shown for the two-pathway models in Figure 3, it is clear that virtually all metabolism at ambient exposure levels is in the low-dose linear range for the high-affinity-pathway two.

### 3.2. Candidates for Two Metabolic Pathways and Implications for Cancer Risks

CYP2E1 is a major enzyme responsible for benzene metabolism in the liver [29,30,31], and we showed previously that genetic variation in this enzyme affected the proportions of benzene metabolites formed at exposure levels above 1 ppm [14], which is equivalent to a predicted value of [*UB*] > 0.23 μM. Thus, it is reasonable to conclude that the low-affinity-pathway one is dominated by hepatic metabolism via CYP2E1 [16]. It follows that human cancer risks derived from dose–response curves involving workers primarily exposed to benzene at air concentrations above 1 ppm would be dominated by hepatic metabolism. Yet, given the very low capacity of high-affinity-pathway two, extrahepatic metabolic enzymes other than CYP2E1 must be involved at sub-ppm exposures. Since the respiratory tract is the site of first contact with airborne toxicants, tissue-specific metabolism represents a concentration-dependent mechanism whereby respiratory enzymes play a predominant role at low-dose exposures, transitioning to hepatic enzymes at high-dose exposures [32]. And if the human respiratory tract is the primary site for this low-dose metabolism of benzene, it follows that cancer risks at ambient exposure levels should be assessed accordingly [16].

In seeking alternatives for the respiratory metabolism of benzene, we focused on two other CYPs, namely CYP2A13 and CYP2F1, as candidate enzymes [16]. Both CYP2A13 and CYP2F1 are preferentially expressed in the human respiratory tract—with little or no expression in other tissues—and efficiently metabolize the aromatic molecule, naphthalene, at low doses [33]. Sheets et al. reported a *K_m_* of 3.83 μM and *V_max_* of 0.01 pmol per pmol CYP/min for CYP2F1 metabolism of benzene, indicating efficient metabolism at nanomolar concentrations in human respiratory cells [34]. Fukami et al. demonstrated that CYP2A13 had higher affinities than CYP2E1 for small aromatic molecules (styrene, toluene, chloroxazone, and *p*-nitrophenol) with reported *K_m_* values that were 7-fold lower than those of CYP2E1 for its marker substrates, chloroxazone (36 vs. 274 μM) and *p*-nitrophenol (5.5 vs. 39.6 μM) [35]. Using CYP2A13/2B6/2F1-transgenic humanized mice, Wei et al. [36] demonstrated that human CYP2A13 and CYP2F1 were highly expressed in respiratory tissues. Immunoblot analysis revealed CYP2A13 protein levels of approximately 100 pmol/mg in nasal mucosa and 0.2 pmol/mg in the lungs. This tissue-specific expression pattern suggests that these CYP genes can be expressed at substantial levels in human respiratory tissues [34]. Thus, the available literature points to efficient metabolism of small aromatic molecules by CYP2A13 and CYP2F1 in the human respiratory tract. We encourage investigators to determine the affinities of these enzymes for benzene specifically relative to that for CYP2E1.

If one or both of the respiratory enzymes CYP2A13 and CYP2F1 contribute to high-affinity-pathway two, then the reported associations of blood cancers with benzene exposures in ambient populations [8,9,10,11] should take this into account. Indeed, there is extensive evidence that CYP2A13 bioactivates the tobacco-specific procarcinogen, 4-(methylnitrosoamino)-1-(3-pyridyl)-1-butanone (NNK), and the procarcinogenic mycotoxin, aflatoxin B1 (AFB1), in either the respiratory tract of humanized mice (NNK) or human bronchial cells (AFB1) [37]. Furthermore, nicotine inhibits the ability of CYP2A13 to bioactivate NNK and AFB1 [37,38,39], consistent with our finding that benzene metabolism via pathway-two is inhibited by smoking in male subjects. The bioactivation of benzene by respiratory CYPs to toxic metabolites would also support evidence that lung-cancer incidence in the general population increases with residential exposure to benzene [1,40] and could potentially contribute to the 25% of lung adenomas observed in never smokers [41].

### 3.3. Other Pathways Affecting Benzene Metabolism

We recognize that benzene metabolism is influenced by multiple saturable processes involving, for example, CYPs, epoxide hydrolases, and GSH transferases (Figure 1) [14]. Interpretation of the MA pathway is simplified because CYPs are the primary enzymes responsible for the metabolism of benzene to benzene oxide-oxepin and for the subsequent oxidation of oxepin to the ring-opened product *E*,*E*-muconaldehyde, followed by MA [42] (see Figure 1). Nonetheless, multiple enzymatic processes open the possibility that some of the dose-related variability in MA metabolism (Figure 2 and Figure 3) could reflect interactions across saturable pathways. Yet, logic would argue that one of the oxidation processes leading to MA would be rate limiting and that interactions with competing pathways would tend to obscure rather than accentuate statistical evidence favoring the two-pathway model for MA.

## 4. Materials and Methods

### 4.1. Study Populations and Biological Monitoring

Subjects were from a cross-sectional study carried out in Tianjin, China where 250 subjects with occupational exposure to benzene (164 females and 86 males) worked in shoe factories where benzene was a component of glues [7]. A group of 139 control workers (87 females and 52 males) were recruited in a nearby clothing factory where only ambient levels of benzene were present. Age, sex, and smoking status were self-reported. A urine specimen was collected from each subject at the end of the work shift and was immediately sealed and subsequently frozen to prevent loss of benzene. Some subjects (*N* = 139) had between two and four urine specimens collected on different days over several months. Subjects had been recruited with informed consent according to protocols that had been approved by institutional review boards at the participating institutions. Urinary benzene and MA were measured in one laboratory by gas-chromatography–mass spectrometry (GC-MS) with experimental details given in [7] along with summary statistics for all subjects stratified by exposure status, sex, age, smoking status, [*UB*], and [*MA*].

### 4.2. Fitting Michaelis–Menten-like Models for Benzene Metabolism

In this investigation, data from [7] were reanalyzed with more extensive models. MM-like models that had previously been fit to predict levels of total urinary metabolites from levels of AB in nonsmoking females [16] were updated to consider relationships between the urinary levels of MA and the corresponding UB concentrations in nonsmoking females (*N* = 243) and, separately, in nonsmoking (*N* = 55) and smoking (*N* = 83) males.

Nonlinear regression models were fit using packages in R (URL https://www.R-project.org/). For subject *i*, each data pair ([*MA*]*_i_* and [*UB*]*_i_*) represents the respective urinary concentrations of MA and UB. For the 139 Chinese subjects with multiple urine specimens, estimated geometric means of [*MA*]*_i_* and [*UB*]*_i_* were used [7].

The kinetic parameters were estimated by optimizing/minimizing the following objective functions for the one-pathway and two-pathway models (Equations (5) and (6), respectively) subject to lower- and upper-bound constraints.

(5) $$F^{\left(1\right)}=\sum_{i}{\left(\ln{\left[MA\right]}_{i}-\ln\left({\left[MA\right]}_{0}^{\left(1\right)}+\frac{V_{max,1}^{\left(1\right)}{\left[UB\right]}_{i}}{\left(K_{m,1}^{\left(1\right)}+{\left[UB\right]}_{i}\right)}\right)\right)}^{2}$$

(6) $$F^{\left(2\right)}=\sum_{i}{\left(\ln{\left[MA\right]}_{i}-\ln\left({\left[MA\right]}_{0}^{\left(2\right)}+\frac{V_{max,1}^{\left(2\right)}{\left[UB\right]}_{i}}{\left(K_{m,1}^{\left(2\right)}+{\left[UB\right]}_{i}\right)}+\frac{V_{max,2}^{\left(2\right)}{\left[UB\right]}_{i}}{\left(K_{m,2}^{\left(2\right)}+{\left[UB\right]}_{i}\right)}\right)\right)}^{2}$$

Specifically the optimization problem solved for the one-pathway model was ${min}_{{\left[MA\right]}_{0}^{\left(1\right)},V_{max,1}^{\left(1\right)},\;K_{m,1}^{\left(1\right)}}F^{\left(1\right)}$, with $0\leq {\left[MA\right]}_{0}^{\left(1\right)}\leq 5$, $0\leq V_{max,1}^{\left(1\right)}\leq 5000,$ and $0\leq K_{m,1}^{\left(1\right)}\leq 1000.$ The optimization problem solved for the two-pathway model was ${min}_{{\left[MA\right]}_{0}^{\left(2\right)},V_{max,1}^{\left(2\right)},\;K_{m,1}^{\left(2\right)},V_{m,2}^{\left(2\right)},\;K_{m,2}^{\left(2\right)}}F^{\left(2\right)}$, with $0\leq {\left[MA\right]}_{0}^{\left(2\right)}\leq 5$, $0\leq V_{max,1}^{\left(2\right)}\leq 5000$, $0\leq K_{m,1}^{\left(2\right)}\leq 1000$, $0\leq V_{max,2}^{\left(2\right)}\leq 5000$, and $0\leq K_{m,2}^{\left(2\right)}\leq 500$.

Let *N* denote the number of subjects in a particular group being modeled. The one-pathway model has three parameters while the two-pathway model has five parameters. Let $F_{opt}^{\left(1\right)}$ and $F_{opt}^{\left(2\right)}$ denote the optimal values for the two optimization problems given above. The optimum/maximum log-likelihood values of the observed [*MA*] values in the logarithmic space are given by $L_{opt}^{\left(1\right)}$ and $L_{opt}^{\left(2\right)}$, where,

(7) $$L_{opt}^{\left(1\right)}=-N\ln\left(\sqrt{\frac{F_{opt}^{\left(1\right)}}{N}}\right)-\frac{N}{2}+N\ln\left(\frac{1}{\sqrt{2\pi }}\right)\;\mathrm{and}$$

(8) $$L_{opt}^{\left(2\right)}=-N\ln\left(\sqrt{\frac{F_{opt}^{\left(2\right)}}{N}}\right)-\frac{N}{2}+N\ln\left(\frac{1}{\sqrt{2\pi }}\right).$$

Values of the Akaike Information Criterion (AIC) for the two models were computed as the following:


(9) $${AIC}^{\left(1\right)}=2\times \left(3-L_{opt}^{\left(1\right)}\right)\;\mathrm{and}$$

(10) $${AIC}^{\left(2\right)}=2\times \left(5-L_{opt}^{\left(2\right)}\right).$$

The *solnp* function that is part of the Rsolnp package in R version 1.16 [Rsolnp: General Non-linear Optimization Using Augmented Lagrange Multiplier Method. R package version 1.16] was used to solve the optimization problems. The optimal solution was assigned to the smallest objective function value among those resulting from 10 random initial starting guesses for the background MA levels and the kinetic parameters. This represented an attempt to avoid locally optimal solutions in favor of globally optimal ones. The initial guesses for ${\left[MA\right]}_{0}^{\left(1\right)}$ and ${\left[MA\right]}_{0}^{\left(2\right)}$ were drawn uniformly from 0.1 to 5. Those for $V_{max,1}^{\left(1\right)}$ and $V_{max,1}^{\left(2\right)}$ were drawn uniformly from 20 to 400, guesses for $K_{m,1}^{\left(1\right)}$ were drawn uniformly from 1 to 20, guesses for $V_{max,2}^{\left(2\right)}$ were drawn uniformly from 1 to 20, and those for $K_{m,2}^{\left(2\right)}$ were drawn uniformly from 0.1 to 2. The bootstrap-based confidence intervals were derived using the percentile method [43] from the parameter estimates using 1000 bootstrapped samples for each of the three groups being studied. The *boot.ci* function that is part of the boot functions in R [boot: Bootstrap R (S-Plus) Functions. R package version 1.3-30] was used for this.

### 4.3. Linear and Generalized Additive Models (GAMs)

The linear models were fit for benzene metabolism using the *lm* function in base R while the GAMs were fit using the *gam* function that is part of the mgcv package in R based on [44]. The spline-based smoothing term for the GAMs was defined using the *s* function of ln[*UB*]. The respective AIC values were derived using the *AIC* function applied on the resulting model fits.

### 4.4. Weights of Evidence for All Models

In judging the weights of evidence favoring the one- and two-pathway MM-like models (Models 1 and 2)—as well as the GAMs and linear models—as depictions of the true metabolism for benzene, we followed lines based on information theory [15] as described in Section 1.3.

## 5. Conclusions

We fit MM-like models having either one or two metabolic pathways to data from 389 Chinese workers to predict the urinary concentrations of MA as functions of UB concentrations that spanned more than a million-fold range. The weight of evidence favoring two pathways was essentially 100 percent for nonsmoking subjects of both sexes and 58 percent for smoking males. The rates of metabolism for the high-affinity-pathway two were much greater than those for the low-affinity-pathway one at ppb air concentrations, particularly in males, and this metabolism was inhibited by smoking. Characteristics of the high-affinity, low-capacity-pathway two are consistent with respiratory metabolism via CYP2A13 and/or CYP2F1, whereas the high-capacity, low-affinity-pathway one primarily represents hepatic metabolism by CYP2E1.

Since virtually all ambient populations are exposed to benzene at air concentrations of less than 10 ppb, these results have several implications regarding potential cancer risks. First, quantitative risk assessment derived from studies of workers exposed to benzene at air concentrations above one ppm likely underestimate risks to the general public by many fold. Second, benzene-derived risks to the general public may be modulated by smoking. And third, the bioactivation of toxic benzene metabolites in the respiratory tract (site of contact) makes the lung a target for cancers in the general public, including never smokers.

## Figures and Tables

**Table 1:** Fit statistics (AIC values, smaller is better) and relative Akaike weights of evidence comparing the two-pathway Model 2 with the one-pathway Model 1, the GAM, and the linear model for female nonsmokers (*N* = 243), male nonsmokers (*N* = 55), and male smokers (*N* = 83).

Model	Female Nonsmokers	Male Nonsmokers	Male Smokers
Linear	605.59	152.83	230.80
GAM	585.29	148.08	212.71
One-path	597.57	156.34	215.98
Two-path	583.84	148.59	215.32
Akaike weight two-path vs. one-path	0.9990	0.9796	0.5820
Akaike weight two-path vs. linear	1.0000	0.8926	0.9996
Akaike weight two-path vs. GAM	0.6737	0.4366	0.2133

**Table 2:** Estimated parameters and 95% confidence intervals (95% CI) for Michaelis–Menten-like models of urinary muconic acid concentrations [*MA*] as functions of the corresponding urinary benzene concentrations [*UB*] in 382 subjects.

	Female NS		Male NS		Male S	
Parameter	Estimate	95% CI	Estimate	95% CI	Estimate	(95% CI)
**Model 1:**						
${\left[MA\right]}_{0}^{\left(1\right)}$	0.973	0.768, 1.18	1.09	0.360, 2.00	0.954	0.655, 1.28
$V_{max,1}^{\left(1\right)}$	78.4	45.0, 138	323	10.3, 500	144	30.6, 541
$K_{m,1}^{\left(1\right)}$	1.27	0.562, 2.80	10.8	0.019, 210	2.54	0.347, 11.6
**Model 2:**						
${\left[MA\right]}_{0}^{\left(2\right)}$	0.735	0.395, 0.952	0.331	0.000, 1.08	0.892	0.613, 1.30
$V_{max,1}^{\left(2\right)}$	170	80.6, 3390	672	0.000, 5000	891	37.7, 5000
$K_{m,1}^{\left(2\right)}$	6.71	1.97, 255	44.7	9.31, 827	66.5	6.38, 1000
$V_{max,2}^{\left(2\right)}$	5.61	1.95, 12.5	4.61	2.46, 449	18.6	7.52, 60.3
$K_{m,2}^{\left(2\right)}$	0.034	0.003, 0.124	0.007	0.003, 18.0	0.280	0.122, 1.19
$Rate\;1=\frac{V_{max,1}^{\left(2\right)}}{\left(K_{m,1}^{\left(2\right)}\right)}$	25.4	12.9, 46.5	15.0	0.000, 28.8	13.4	1.99, 31.1
$Rate\;2=\frac{V_{max,1}^{\left(2\right)}}{\left(K_{m,1}^{\left(2\right)}\right)}$	165	82.2, 692	642	24.2, 1260	66.2	29.9, 114
*Rate 2/Rate 1*	6.50	2.87, 20.1	42.7	9.33, 6.8 × 10^8^	4.94	1.06, 40.2
*Rate 2 proportion*	0.867	0.742, 0.952	0.977	0.903, 1.00	0.831	0.514, 0.975

Legend: NS, nonsmokers; S, smokers. Model 1 has one metabolic pathway and Model 2 has two metabolic pathways; units for ${\left[MA\right]}_{0}$ and $V_{max}\;$are (μM MA) and for $K_{m}$ are (μM UB); *Rate 1* and *Rate 2* are the maximum rates of low-dose benzene metabolism for pathway-one and pathway-two, respectively, and $Rate\;2\;proportion=\frac{Rate\;2}{Rate\;1+Rate2}$ is the proportion of low-dose metabolism under pathway-two.

## Data Availability

The data used for these analyses are provided in Supplementary Materials as supplementary data.xlsx. They represent a subset of the dataset originally analyzed by Kim et al. [7,13].

## Supplementary Materials

The following supporting information can be downloaded at https://www.mdpi.com/article/10.3390/ijms26178550/s1.

## Abbreviations

AB, air-benzene concentration; ΔAIC*_i_*, the difference in AIC values between the *i*th model and the better model; AIC, Akaike’s Information Criterion; DSM, dose-specific metabolism; *w*, Akaike weight for a given model; MA, *E-E*-muconic acid; [*MA*], urinary concentration of MA (μM); [*MA*]_0_, background concentration of MA (μM); ppb, parts per billion by volume in air; ppm, parts per million by volume in air; UB, urinary benzene; [*UB*], urinary benzene concentration (μM).

## References

[1] IARC (2018). IARC Monographs on the Evaluation of Carcinogenic Risks to Humans: Benzene.

[2] Glass D.C., Gray C.N., Jolley D.J., Gibbons C., Sim M.R., Fritschi L., Adams G.G., Bisby J.A., Manuell R. (2003). Leukemia risk associated with low-level benzene exposure. Epidemiology.

[3] Linet M.S., Yin S.N., Gilbert E.S., Dores G.M., Hayes R.B., Vermeulen R., Tian H.Y., Lan Q., Portengen L., Ji B.T. (2015). A retrospective cohort study of cause-specific mortality and incidence of hematopoietic malignancies in Chinese benzene-exposed workers. Int. J. Cancer.

[4] Snyder R. (2004). Xenobiotic metabolism and the mechanism(s) of benzene toxicity. Drug Metab. Rev..

[5] Ross D. (2000). The role of metabolism and specific metabolites in benzene-induced toxicity: Evidence and issues. J. Toxicol. Environ. Health A.

[6] De Palma G., Manno M. (2014). Metabolic polymorphisms and biomarkers of effect in the biomonitoring of occupational exposure to low-levels of benzene: State of the art. Toxicol. Lett..

[7] Kim S., Vermeulen R., Waidyanatha S., Johnson B.A., Lan Q., Rothman N., Smith M.T., Zhang L., Li G., Shen M. (2006). Using urinary biomarkers to elucidate dose-related patterns of human benzene metabolism. Carcinogenesis.

[8] Teras L.R., Diver W.R., Deubler E.L., Krewski D., Flowers C.R., Switchenko J.M., Gapstur S.M. (2019). Residential ambient benzene exposure in the United States and subsequent risk of hematologic malignancies. Int. J. Cancer.

[9] Schnatter A.R., Glass D.C., Tang G., Irons R.D., Rushton L. (2012). Myelodysplastic syndrome and benzene exposure among petroleum workers: An international pooled analysis. J. Natl. Cancer Inst..

[10] Carlos-Wallace F.M., Zhang L., Smith M.T., Rader G., Steinmaus C. (2016). Parental, In Utero, and Early-Life Exposure to Benzene and the Risk of Childhood Leukemia: A Meta-Analysis. Am. J. Epidemiol..

[11] Filippini T., Hatch E.E., Rothman K.J., Heck J.E., Park A.S., Crippa A., Orsini N., Vinceti M. (2019). Association between Outdoor Air Pollution and Childhood Leukemia: A Systematic Review and Dose-Response Meta-Analysis. Environ. Health Perspect..

[12] Rappaport S.M., Waidyanatha S., Qu Q., Shore R., Jin X., Cohen B., Chen L.C., Melikian A.A., Li G., Yin S. (2002). Albumin adducts of benzene oxide and 1,4-benzoquinone as measures of human benzene metabolism. Cancer Res..

[13] Kim S., Vermeulen R., Waidyanatha S., Johnson B.A., Lan Q., Smith M.T., Zhang L., Li G., Shen M., Yin S. (2006). Modeling human metabolism of benzene following occupational and environmental exposures. Cancer Epidemiol. Biomarkers Prev..

[14] Kim S., Lan Q., Waidyanatha S., Chanock S., Johnson B.A., Vermeulen R., Smith M.T., Zhang L., Li G., Shen M. (2007). Genetic polymorphisms and benzene metabolism in humans exposed to a wide Range of air concentrations. Pharmacogenet Genom..

[15] Burnham K.P., Anderson D.R. (2002). Model Selection and Multimodel Inference: A Practical Information-Theoretic Approach.

[16] Rappaport S.M., Kim S., Lan Q., Vermeulen R., Waidyanatha S., Zhang L., Li G., Yin S., Hayes R.B., Rothman N. (2009). Evidence that humans metabolize benzene via two pathways. Environ. Health Perspect..

[17] Price P.S., Rey T.D., Fontaine D.D., Arnold S.M. (2012). A reanalysis of the evidence for increased efficiency in benzene metabolism at airborne exposure levels below 3 p.p.m. Carcinogenesis.

[18] Rappaport S.M., Kim S., Thomas R., Johnson B.A., Bois F.Y., Kupper L.L. (2013). Low-dose metabolism of benzene in humans: Science and obfuscation. Carcinogenesis.

[19] Price P.S., Rey T.D., Fontaine D.D., Arnold S.M. (2013). Letter to the editor in response to ‘Low-dose metabolism of benzene in humans: Science and obfuscation’ Rappaport et al. (2013). Carcinogenesis.

[20] Rappaport S.M., Johnson B.A., Bois F.Y., Kupper L.L., Kim S., Thomas R. (2013). Ignoring and adding errors do not improve the science. Carcinogenesis.

[21] McNally K., Sams C., Loizou G.D., Jones K. (2017). Evidence for non-linear metabolism at low benzene exposures? A reanalysis of data. Chem. Biol. Interact..

[22] Cox L.A., Schnatter A.R., Boogaard P.J., Banton M., Ketelslegers H.B. (2017). Non-parametric estimation of low-concentration benzene metabolism. Chem. Biol. Interact..

[23] Rappaport S.M., Kim S., Lan Q., Li G., Vermeulen R., Waidyanatha S., Zhang L., Yin S., Smith M.T., Rothman N. (2010). Human benzene metabolism following occupational and environmental exposures. Chem. Biol. Interact..

[24] Kettl S. (1991). Accounting for heteroscedasticity in the transform both sides regression model. J. R. Stat. Soc. Ser. C Appl. Stat..

[25] Ruppert T., Scherer G., Tricker A.R., Adlkofer F. (1997). trans,trans-muconic acid as a biomarker of non-occupational environmental exposure to benzene. Int. Arch. Occup. Environ. Health.

[26] Leung P.L., Harrison R.M. (1998). Evaluation of personal exposure to monoaromatic hydrocarbons. Occup. Environ. Med..

[27] Hoffmann K., Krause C., Seifert B., Ullrich D. (2000). The German Environmental Survey 1990/92 (GerES II): Sources of personal exposure to volatile organic compounds. J. Expo. Anal. Environ. Epidemiol..

[28] Wallace L.A. (1987). The Total Exposure Assessment Methodology (TEAM) Study: Summary and Analysis: Volume I.

[29] Nedelcheva V., Gut I., Soucek P., Tichavska B., Tynkova L., Mraz J., Guengerich F.P., Ingelman-Sundberg M. (1999). Metabolism of benzene in human liver microsomes: Individual variations in relation to CYP2E1 expression. Arch. Toxicol..

[30] Valentine J.L., Lee S.S.T., Seaton M.J., Asgharian B., Farris G.M., Corton J.C., Gonzalez F.J., Medinsky M.A. (1996). Reduction of benzene metabolism and toxicity in mice that lack CYP2E1 expression. Toxicol. Appl. Pharmacol..

[31] Powley M.W., Carlson G.P. (2000). Cytochromes P450 involved with benzene metabolism in hepatic and pulmonary microsomes. J. Biochem. Mol. Toxicol..

[32] Ding X., Kaminsky L.S. (2003). Human extrahepatic cytochromes P450: Function in xenobiotic metabolism and tissue-selective chemical toxicity in the respiratory and gastrointestinal tracts. Annu. Rev. Pharmacol. Toxicol..

[33] Li L., Carratt S., Hartog M., Kovalchik N., Jia K., Wang Y., Zhang Q.Y., Edwards P., Winkle L.V., Ding X. (2017). Human CYP2A13 and CYP2F1 Mediate Naphthalene Toxicity in the Lung and Nasal Mucosa of CYP2A13/2F1-Humanized Mice. Environ. Health Perspect..

[34] Sheets P.L., Yost G.S., Carlson G.P. (2004). Benzene metabolism in human lung cell lines BEAS-2B and A549 and cells overexpressing CYP2F1. J. Biochem. Mol. Toxicol..

[35] Fukami T., Katoh M., Yamazaki H., Yokoi T., Nakajima M. (2008). Human cytochrome P450 2A13 efficiently metabolizes chemicals in air pollutants: Naphthalene, styrene, and toluene. Chem. Res. Toxicol..

[36] Wei Y., Wu H., Li L., Liu Z., Zhou X., Zhang Q.Y., Weng Y., D’Agostino J., Ling G., Zhang X. (2012). Generation and characterization of a CYP2A13/2B6/2F1-transgenic mouse model. Drug Metab. Dispos..

[37] Vrzal R. (2021). Genetic and Enzymatic Characteristics of CYP2A13 in Relation to Lung Damage. Int. J. Mol. Sci..

[38] von Weymarn L.B., Brown K.M., Murphy S.E. (2006). Inactivation of CYP2A6 and CYP2A13 during nicotine metabolism. J. Pharmacol. Exp. Ther..

[39] Liu X., Zhang J., Zhang C., Yang B., Wang L., Zhou J. (2016). The inhibition of cytochrome P450 2A13-catalyzed NNK metabolism by NAT, NAB and nicotine. Toxicol. Res..

[40] Chiavarini M., Rosignoli P., Sorbara B., Giacchetta I., Fabiani R. (2024). Benzene Exposure and Lung Cancer Risk: A Systematic Review and Meta-Analysis of Human Studies. Int. J. Environ. Res. Public Health.

[41] LoPiccolo J., Gusev A., Christiani D.C., Janne P.A. (2024). Lung cancer in patients who have never smoked—An emerging disease. Nat. Rev. Clin. Oncol..

[42] Bleasdale C., Cameron R., Edwards C., Golding B.T. (1997). Dimethyldioxirane converts benzene oxide/oxepin into (Z,Z)-muconaldehyde and sym-oxepin oxide: Modeling the metabolism of benzene and its photooxidative degradation. Chem. Res. Toxicol..

[43] Effron B., Tibshirani R. (1994). An Introduction to the Bootstrap.

[44] Wood S.N. (2011). Fast stable restricted maximum likelihood and marginal likelihood estimation of semiparametric generalized linear models. J. R. Stat. Soc..

