# Supplementary material for: Benzene Metabolism Is Dominated by a High-Affinity Pathway at Ambient Exposures with Implications for Cancer Risks
Source: Int J Mol Sci. 2025 Sep 3;26(17):8550. doi: 10.3390/ijms26178550 (PMC12428784; doi:10.3390/ijms26178550)
Supplement: Supplementary file 1 [file ijms-26-08550-s001.zip › figures.pdf]

Supplementary Figures

Benzene metabolism is dominated by a high-affinity pathway at ambient exposures with implications for cancer risks

Authors: Reuben Thomas, Sungkyoon Kim, Qing Lan, Roel Vermeulen, Luoping Zhang, Nathaniel Rothman, Martyn T. Smith and Stephen M. Rappaport

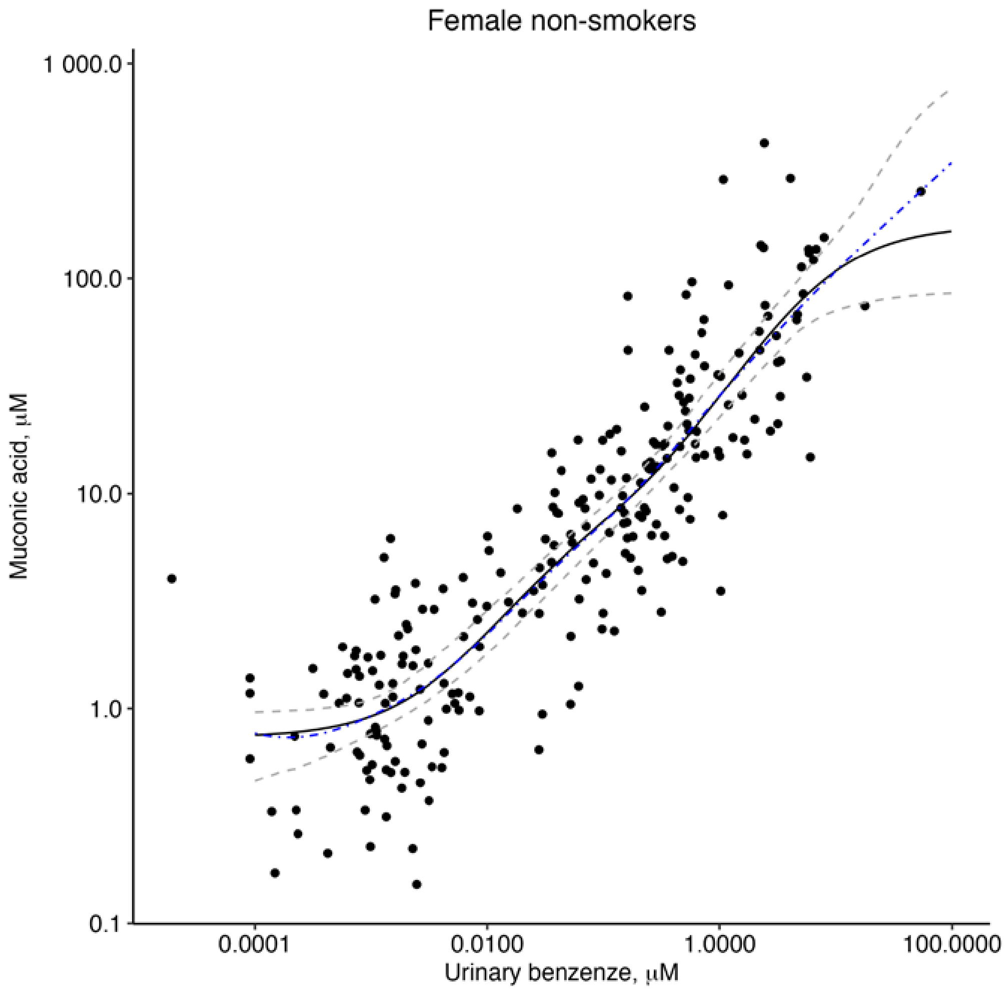

Figure S1. Comparison of predicted relationships of urinary muconic acid levels versus urinary benzene levels for female nonsmokers. The black line represents the two-pathway Michaelis-Menten model (with grey dashed lines representing the 95% confidence interval) and the blue dashed line represents the general additive model.

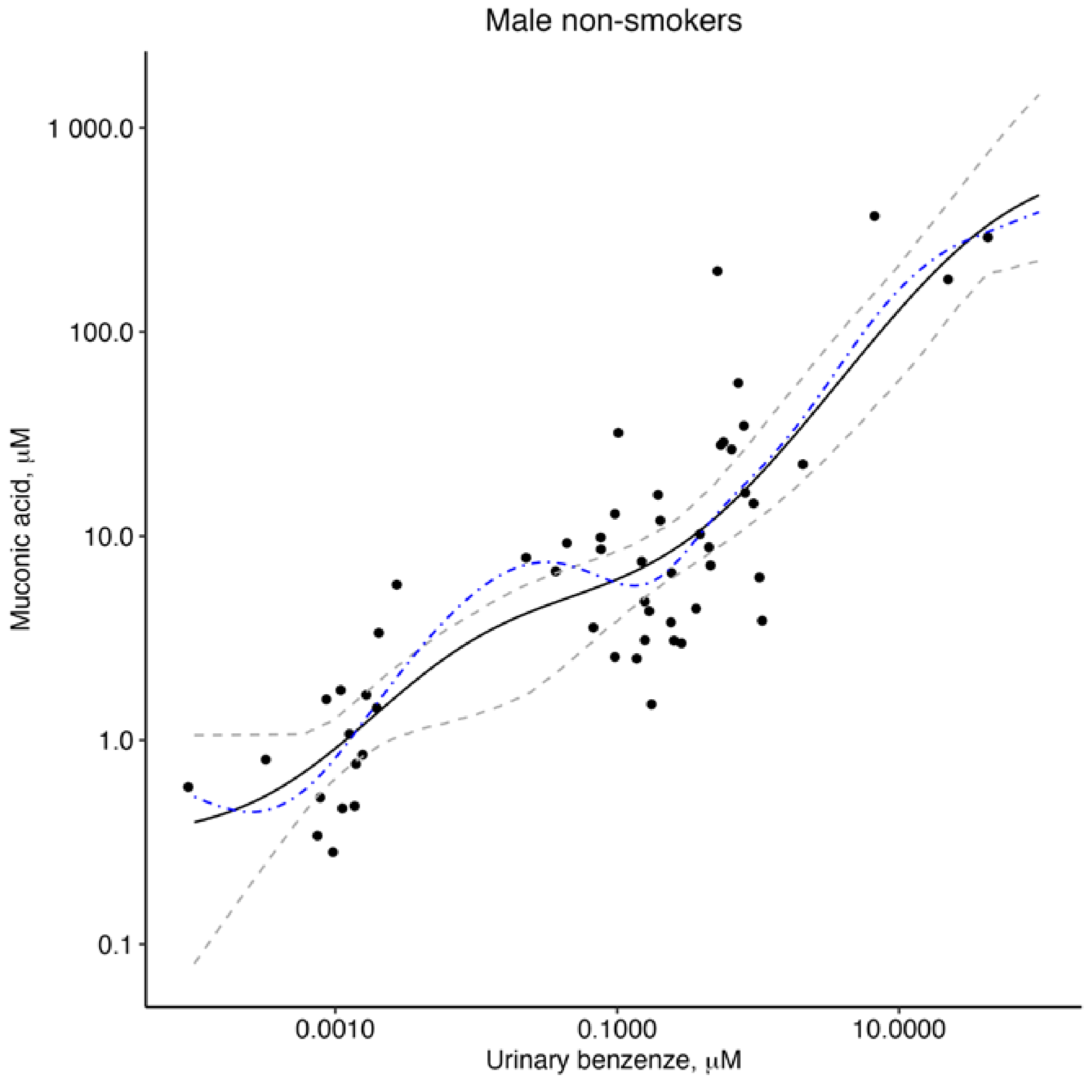

Figure S2. Comparison of predicted relationships of urinary muconic acid levels versus urinary benzene levels for male nonsmokers. The black line represents the two-pathway Michaelis-Menten model (with grey dashed lines representing the 95% confidence interval) and the blue dashed line represents the general additive model.

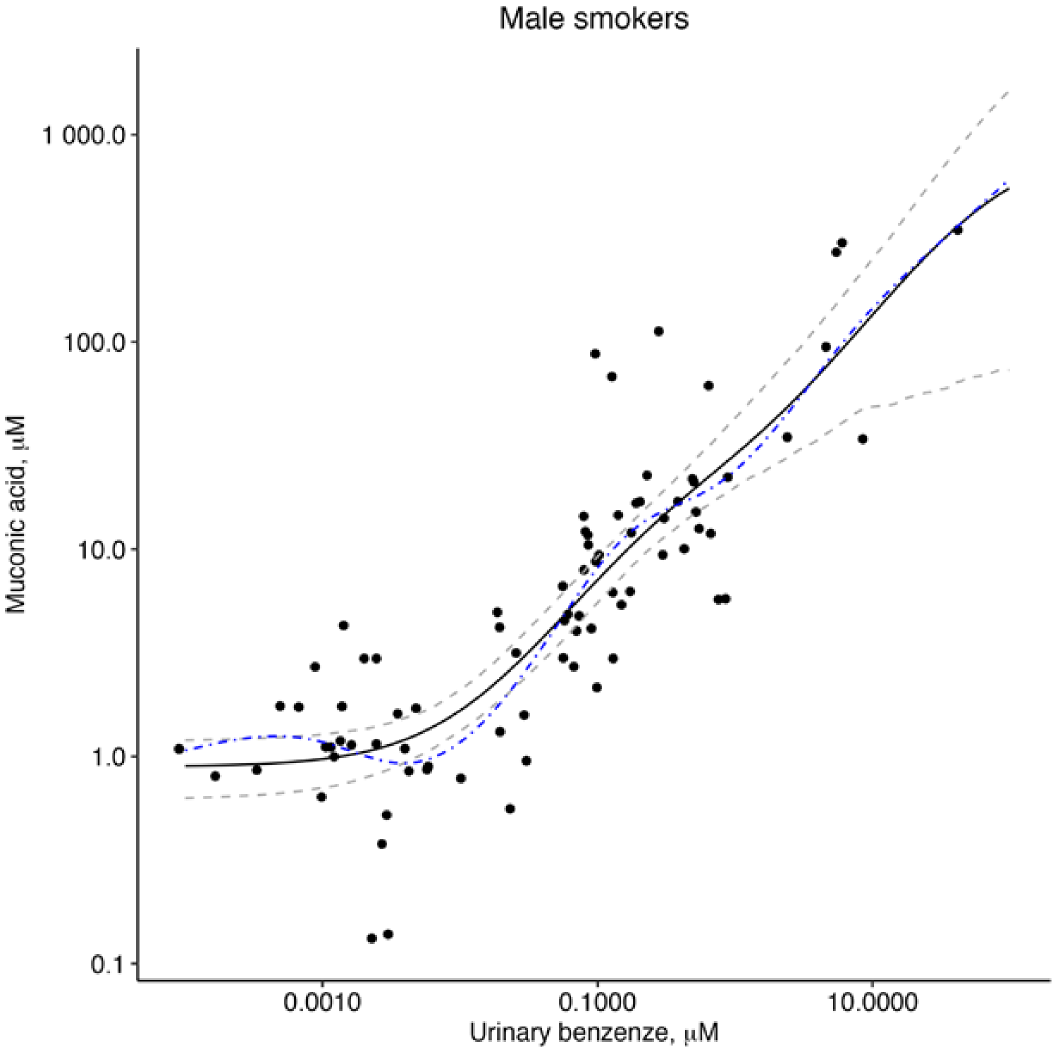

Figure S3. Comparison of predicted relationships of urinary muconic acid levels versus urinary benzene levels for male smokers. The black line represents the two-pathway Michaelis-Menten model (with grey dashed lines representing the 95% confidence interval) and the blue dashed line represents the general additive model.
